# Association Between Sleep Disturbance and Behavioral Feeding Problems in Children and Adolescents with Autism Spectrum Disorder

**DOI:** 10.3390/diseases13090280

**Published:** 2025-08-29

**Authors:** Milagros Fuentes-Albero, Mayra Alejandra Mafla-España, José Martínez-Raga, Omar Cauli

**Affiliations:** 1 Ripalda Clinic, 46001 Valencia, Spain; milafuentes@clinicaripalda.es; 2 Department of Nursing, University of Valencia, 46010 Valencia, Spain; maymaes@alumni.uv.es; 3 Department of Psychiatry and Clinical Psychology, Hospital Universitario Doctor Peset, University of Valencia, 46010 Valencia, Spain; jose.martinez-raga@uv.es

**Keywords:** sleep disorders, eating disorders, autism spectrum disorder

## Abstract

Introduction: Children and adolescents with autism spectrum disorder (ASD) often present sleep and eating problems. However, the relationship between these two factors has seldom been studied. Objective: This paper aimed to examine the association between sleep disturbances and feeding problems in children and adolescents with ASD. Methods: This cross-sectional observational study assessed feeding behaviors using the Behavioral Pediatrics Feeding Assessment Scale (BPFAS) and assessed sleep problems with the Sleep Disturbance Scale for Children (Bruni scale). Bivariate analyses and multivariate logistic and linear regression analyses were performed. Results: Sleep disturbances were significantly associated with autism severity (*p* = 0.003), but not with BPFAS subscale scores. Multivariate logistic regression indicated that sleep disturbances were independently associated with autism severity (*p* = 0.01; OR = 0.23; 95% CI: 0.06–0.77) and the BPFAS frequency subscale score (*p* = 0.01; OR = 1.04; 95% CI: 1.01–1.07). A secondary logistic regression identified five BPFAS items significantly associated with sleep disturbances: difficulty chewing (*p* = 0.02, OR = 0.12, 95% CI 0.02–0.74), voluntary attendance at meals (*p* = 0.01, OR = 0.60, 95% CI 0.39–0.90), tantrums during meals (*p* < 0.001; OR = 2.08, 95% CI 1.21–3.56), poor appetite (*p* < 0.001; OR = 2.63, 95% CI 1.43–4.82), and the caregiver’s perception that the child’s eating habits negatively affected their health (*p* = 0.03; OR = 1.53, 95% CI 1.03–2.40). No significant associations were found with age, sex, medical comorbidities, behavioral disorders or genetic factors. Conclusions: The findings suggest that greater autism severity and more pronounced feeding behaviors are independently associated with an increased risk of sleep disturbances in children and adolescents with ASD. Specific maladaptive mealtime behaviors, such as poor appetite, tantrums, and chewing difficulties, may serve as predictors of sleep problems, highlighting the need for integrated screening and early intervention strategies.

## 1. Introduction

Autism spectrum disorder (ASD) is a neurodevelopmental disorder that emerges in early childhood and persists throughout the lifespan. It is characterized by a heterogenous combination of impairments in social communication and social interaction, stereotyped and/or repetitive behaviors, sensory processing abnormalities, and restricted interests [1]. Recent global estimates suggest that ASD affects 1% of the population worldwide [2]. The majority of individuals with autism have significantly increased rates of other medical or psychiatric conditions, including gastrointestinal problems, epilepsy and other neurological disorders, depression, anxiety, attention-deficit/hyperactivity disorder (ADHD), feeding disorders, and sleep disorders [3,4]. These co-occurring disorders often complicate the diagnostic process and can exacerbate core ASD symptoms and impact the quality of life and functioning of affected individuals and their families [5,6].

Sleep disorders in ASD are common in individuals with ASD. Between 40 and 80% of children and adolescents with ASD have sleep problems; however, only about 30% receive a diagnosis of a sleep disorder [7,8,9]. In the overall pediatric population, approximately 40% of children have sleep disturbances, but these are often resolved with age [10]. In contrast, sleep problems in individuals with ASD tend to persist into adulthood [11]. Typically, they include difficulties with sleep onset, circadian sleep disturbances, frequent and prolonged night awakenings, irregular sleep–wake patterns, reduced total sleep duration, and early-morning waking, and worse sleep quality [8]. Sleep disorders in ASD are associated with increased daytime behavioral problems, including self-harm, irritability, aggressive behaviors, low frustration tolerance, and social withdrawal [12,13], and they negatively impact both quality of life and physical health [5,6]. Sleep disorders may also exacerbate core autism symptoms, thereby reducing global functioning [14]. Furthermore, these problems often affect the mental health of caregivers [15,16,17].

The etiology of sleep disturbances in ASD is typically multifactorial. The complex interaction of biological factors, such as genetic vulnerability [18], melatonin dysregulation [19,20,21], or circadian rhythm disorders (e.g., delayed sleep phase disorder and irregular sleep–wake patterns) [22], altered sensory sensitivities (e.g., heightened sensitivity to noise, light, or textures) [23,24], anxiety, stress and hyperarousal (e.g., generalized anxiety, bedtime or racing thoughts) [25,26], and behavioral challenges, including rigid routines and difficulties with transitions, particularly those related to stereotypical behavior [27].

Feeding disorders are highly prevalent, affecting nearly 70% of individuals with ASD [28]. Common feeding challenges include restrictive diets (e.g., refusal of new foods, limited food repertoire, food aversions, food texture preferences, or sensory overload) [29,30,31,32,33], disruptive behaviors during meals (e.g., tantrums, leaving the table, or ritualistic eating), atypical feeding behaviors (e.g., pica, vomiting, regurgitation, or rapid eating) [32,34], and avoidant/restrictive food intake disorder (ARFID), a condition characterized by persistent insufficient nutritional due to low interest in eating, avoidant eating behavior based on sensory characteristics of foods (sensory limitations), and/or concern about the aversive consequences of eating [35]. These feeding challenges may lead to vitamin deficiencies [36], obesity [37], disruptive behaviors [38], and elevated family stress [33,39,40]. Feeding difficulties are often linked to underlying sensory sensitivities, such as oral hypersensitivity, behavioral rigidity, and impaired interoception [31,38].

Children with ASD exhibit elevated rates of sleep disturbances and gastrointestinal symptoms [41,42], which can have a detrimental effect on the quality of life of affected children and their carers [40]. Recent studies have evidenced an association of gastrointestinal problems with sleep disorders and behavioral challenges in autism [43,44].

Some of the characteristics associated with autism, such as impaired sensory processing, can influence the development of feeding disorders like food selectivity [29], and can also contribute to problems of falling and staying asleep (temperature, bedding texture, etc.) [24]. Cognitive rigidity can affect sleeping and feeding habits [34,45].

At the neurobiological level, altered melatonin synthesis leads to sleep disorders and changes in appetite regulation. Serotonin, the precursor neurotransmitter of melatonin, is also altered in autism, and is associated with restrictive eating behaviors and sleep disturbances [46,47]. A possible orexin deficiency has also been linked to selective feeding and sleep problems in autism [48,49].

Despite common etiopathogenic mechanisms, the relationship between sleep disorders and eating disorders is underexplored at the clinical level. The purpose of this study is to study this relationship at the clinical level, through the symptoms observed by parents, in order to design common therapeutic tools in the future that improve the quality of life of people with autism and these comorbidities. The present study aims to examine whether a correlation exists between sleep problems and feeding disorders in ASD. We also want to determine whether this relationship is influenced by variables, such as the severity of autism, age, sex, etc., in order to improve clinical understanding of these comorbidities.

## 2. Materials and Methods

### 2.1. Study Design and Population

A cross-sectional observational study was conducted based on convenience sampling, involving children and adolescents with a prior diagnosis of autism spectrum disorder (ASD). The diagnosis and severity of ASD were confirmed by a Child and Adolescent Psychiatrist, according to the Diagnostic and Statistical Manual of Mental Disorders, Fifth Edition (DSM-5) criteria. Relevant information was collected in interviews with parents or caregivers, conducted during routine consultations with the specialist. To calculate the sample size, we hypothesize a priori a moderate association between sleep disturbances and feeding behavioral problems with a correlation coefficient of r = 0.4; a two-tailed hypothesis, an error of α = 0.05 with a confidence interval of 95% and β error = 20%, and power analysis of 1 − β = 0.80 were also considered. A drop-out rate due to incompleteness of the questionnaires of 10% was anticipated. Since correlation coefficient values below 0.3 are considered to be weak, values 0.3–0.7 are moderate and >0.7 are strong [50,51]; in order to calculate sample size, we assumed a moderate value of the coefficient correlation of 0.4. which led to 52 subjects. The ARCSINUS approximation (GRANMO Sample Size Calculator v.8 available at https://www.datarus.eu/aplicaciones/granmo/ (accessed on 1 February 2025)) was used. A posteriori statistical power led to 83% power, which was a good study result in terms of power (https://clincalc.com/stats/power.aspx (accessed on 25 July 2025)).

Feeding habits were measured using the Behavioral Pediatrics Feeding Assessment Scale (BPFAS) and sleep quality was measured using the Sleep Disturbance Scale for Children through a survey administered to parents visiting the Clinic Ripalda (Valencia, Spain) via an ad hoc questionnaire in Google Forms. The study took place during the months of February–April 2025. Informed consent was included in the form itself, and it had to be accepted in order to access the survey. The Child and adolescent Psychiatrist (Dr. Fuentes-Albero) sent invitations through email to all caregivers of children and adolescents with ASD attending the Clinical Ripalda Medical Center in order to take part to the survey. Informed consent could be revoked by notifying the principal researcher by email or phone call. The study protocol was approved by the Ethics Research Committee of the University of Valencia (Valencia, Spain) (protocol number H1397475950160).

### 2.2. Behavioral Pediatric Feeding Assessment Scale (BPFAS) and Sleep Disturbance Scale for Children (SDSC Bruni Disturbance Sleep Scale)

The BPFAS, developed by Crist and Napier-Phillips in 2001 [52], is an instrument designed to assess mealtime behaviors in young children associated with poor nutritional intake, based on parents responses. The scale consists of 35 items: the first 25 directly assess the child’s feeding behaviors, while the remaining 10 capture parents’ perceptions, feelings, and strategies for addressing these behaviors. Each item is answered using a five-point Likert-type scale, ranging from “never” to “always,” and the parents are also asked to indicate whether they consider the behavior to be problematic (“yes” or “no”). The scale has been validated in Spanish [53]. A cut-off point of ≥84 was established on the frequency subscale to indicate a significant disturbance in feeding behavior, and a cut-off point of ≥9 on the parental perception subscale reflected greater concern or conflict reported by caregivers.

The Sleep Disturbance Scale for Children (SDSC) is a parent-reported screening instrument designed to identify the frequency of sleep disturbances over the past six months in children and adolescents aged 3–16 years [54]. It consists of 26 items organized into six subscales: disturbed sleep onset and maintenance (DIMS, 7 items), sleep-disordered breathing (SBD, 3 items), disturbed arousal (DA, 3 items), disturbed sleep–wake transition (SWTD, 6 items), excessive daytime sleepiness (DOES, 5 items), and sleep hyperhidrosis (SHY, 2 items). Each item is scored on a 5-point Likert scale (1 = never to 5 = always), and the total score ranges from 26 to 130, with higher scores indicating greater sleep disturbance. The clinical cut-off point is ≥39. This scale has been validated in Spanish demonstrating adequate psychometric properties [55].

### 2.3. Statistical Analysis

For the statistical analysis, quantitative variables were reported using the arithmetic mean and standard error of the mean (SEM), and range, while qualitative variables were reported using percentages. The normality of the variables was assessed using the Kolmogorov–Smirnov test. Since the assumption of normality was not met for any of the quantitative variables, non-parametric statistical tests were applied, including the Mann–Whitney U test for comparisons between two independent groups (for example, children/adolescents with or without sleep disturbances), and the Kruskal–Wallis test for comparisons between more than two groups (for example, when comparing the scores of sleep disturbance scale or feeding behavior among children/adolescents with I, II, and III grade severity of ASD). Correlations between continuous variables were analyzed with Spearman’s coefficient, and associations between categorical variables were assessed using Pearson’s chi-square test. Multivariate analyses using binary logistic regression were performed to identify variables associated with the presence or absence of sleep disorders based on the cut-off score of the SDSC scale. The independent variables included in the model were age, sex, autism spectrum disorder severity, presence of associated medical conditions, genetic disorders, and scores on the BPFAS frequency and parent-perceived problems subscales. A second logistic regression was performed in order to identify the items of the SDSC scale that significantly associated with the presence or absence of sleep disorders based on the cut-off score of the SDSC scale. The Cox and Snell R2 and the Nagelkerke R2 in order to estimate the explanatory power of the logistic regression model.

We also calculated collinearity among predictors in the regression models by measuring the variance inflation factor (VIF) and its reciprocal tolerance. These statistics are based on the R-squared value obtained by regressing a predictor on all of the other predictors in the regression analysis. A VIF of 1 means that there is no correlation among a predictor and the remaining predictor variables, and hence the variance of estimated regression coefficient is not inflated at all. SPSS version 28.0 (SPSS, Inc., Chicago, IL, USA) was used throughout the study. A *p* value < 0.05 was considered statistically significant.

## 3. Results

### 3.1. Clinical and Sociodemographic Characteristics

A total of 96 children and adolescents diagnosed with ASD were included in this study. The mean age was 10.73 ± 0.41 years (SEM), ranging from 3 to 17 years. According to DSM-5 criteria, 36.5% (n = 35) were classified as level I autism, 34.4% (n = 33) as level II autism, and 29.2% (n = 28) as level III autism. Medical comorbidities were present in 55.2% of participants (n = 53) while 44.8% (n = 43) reported none. Genetic abnormalities were identified in 19.8% (n = 19) of the sample, while 80.2% (n = 77) had no genetic disorder. Furthermore, psychiatric comorbidities were present in 65.6% (n = 63) and absent in 34.4% (n = 33). The complete data are shown in Table 1.

### 3.2. Feeding Behavior Assessment in Children and Adolescents with ASD

On the BPFAS frequency subscale, the mean score was 59.6 ± 1.69 (SEM) (range: 26–105), and on the parent-perceived problems subscale, the mean score was 2.20 ± 0.53 (SEM) (range: 0–27). Using cut-off score of ≥ 84 points for the frequency subscale, 12.5% of the sample (n = 12) met the criteria for significant feeding disturbances, while 87.5% (n = 84) did not. Regarding caregiver-perceived problems (cut-off score ≥ 9), 89.6% (n = 86) reported no feeding difficulties, while 10.4% (n = 10) reported problematic feeding behaviors in their children.

**Figure 1:**
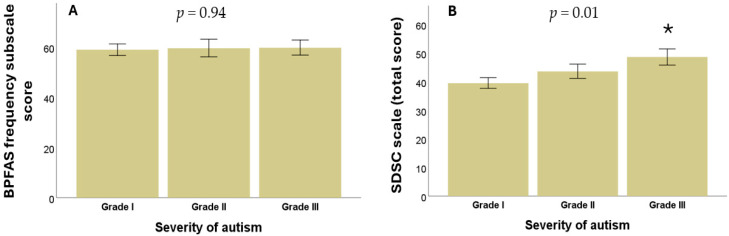
(**A**) Relationship between ASD severity and BPFAS frequency subscale scores. (**B**) Relationship between the severity of ASD and the presence of sleep disorders in children and adolescents. * means a significant difference.

Non-parametric statistical analyses were performed to evaluate the relationship between autism severity according to DSM-5 levels, scores on the frequency subscale, and the parent-perceived problems subscale on the BPFAS. No statistically significant differences were observed between ASD severity levels and BPFAS frequency subscale scores (*p* = 0.94), as shown in Figure 1A, or the parent-perceived problems subscale (*p* = 0.29, Kruskal–Wallis test in all cases).

### 3.3. Sleep Disturbances in Children and Adolescents with ASD

The mean total score on the SDSC (Bruni Sleep Disorders Scale for Children) was 43.6 ± 13.7 (SEM), with a range of 26 to 90 points. The mean scores in each of the SDSC subscales were as follows: DIMS 13.5 ± 4.7 (SEM) (range: 7–28); SBD 4.6 ± 2.7 (SEM) (range: 3–15); DA 3.7 ± 1.4 (SEM) (range: 3–9); SWTD 9.7 ± 3.0 (SEM) (range: 6–22); DOES 8.5 ± 3.9 (SEM) (range: 5–21); and SHY 3.3 ± 2.0 (SEM) (range: 2–10). Based on the cut-off score of ≥39 for poor sleep quality, 52.1% (n = 50) of participants had sleep disorders, while 47.9% (n = 46) did not.

Bivariate analyses were conducted to examine the relationship between ASD severity level and both the total score in the SDSC scale and each of its six subscales. Statistically significant differences were found between autism severity levels and the total Bruni score (*p* = 0.01), as shown in Figure 1B, as well as in two specific subscales: DIMS (*p* = 0.003) and DOES (*p* = 0.01). In contrast, no significant differences were observed between autism severity for the SBD (*p* = 0.15), DA (*p* = 0.59), SWTD (*p* = 0.27), or SHY (*p* = 0.50) (Kruskal–Wallis test in all cases).

### 3.4. Relationship Between Sleep Disorders and Eating Problems in Children and Adolescents with ASD

**Figure 2:**
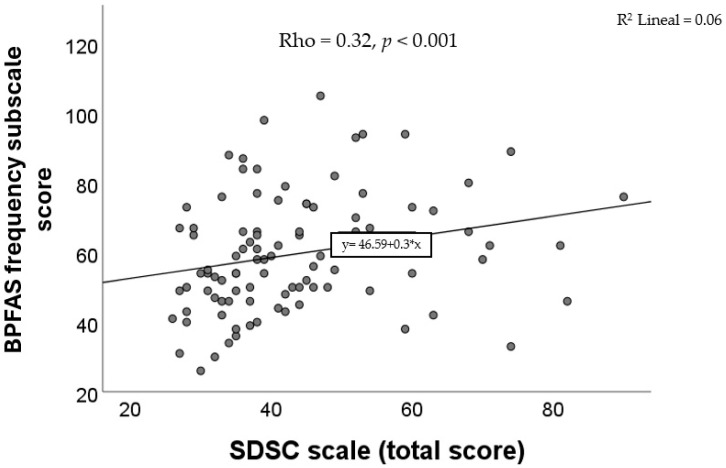
Correlation between the score of SDSC and the score of the frequency subscale of the BPFAS.

Positive and statistically significant correlations were observed between the SDSC total score and the BPFAS frequency subscale (Rho = 0.32, *p* < 0.001), as shown in Figure 2. Likewise, significant associations were also identified between four of the SDSC subscale scores and the BPFAS frequency score: DIMS (Rho = 0.33, *p* < 0.001), DA (Rho = 0.23, *p* = 0.02), SWTD (Rho = 0.22, *p* = 0.02), and DOES (Rho = 0.21, *p* = 0.03). No significant correlations were found between the BPFAS frequency score and the SBD (Rho = 0.05, *p* = 0.62) or SHY (Rho = 0.15, *p* = 0.13, Spearman’s correlation in all cases).

There were no significant correlations between the total score on the SDSC scale and the total score of the parent-perceived problems subscale of the BPFAS (Rho = 0.10, *p* = 0.31). Likewise, there were no significant correlations between each of the SDSC subscales: DIMS (Rho = 0.16, *p* = 0.10), SBD (Rho = −0.17, *p* = 0.08), DA, (Rho = 0.05, *p* = 0.59), SWTD (Rho = 0.09, *p* = 0.33), DOES (Rho = 0.03, *p* = 0.73), SHY (Rho = −0.004, *p* = 0.96, Spearman’s correlation in all cases).

**Figure 3:**
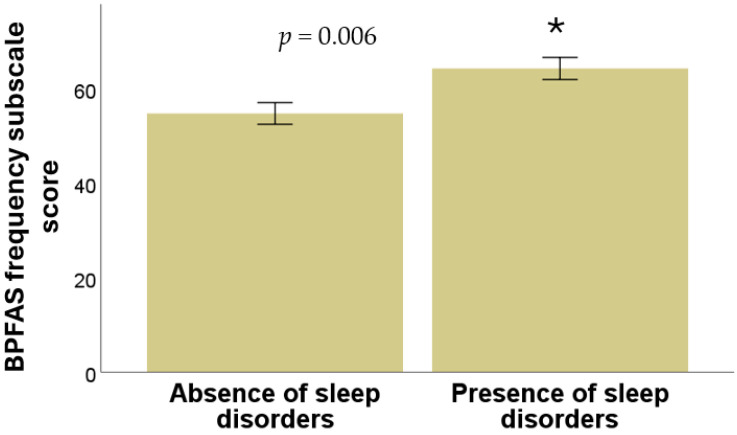
Relationship between the presence and absence of sleep disorders with the score of the frequency subscale of the BPFAS questionnaire. * means a significant difference.

Significant difference was found between the presence or absence of sleep disorders and the frequency subscale score of the BPFAS (*p* = 0.006), as shown in Figure 3. However, no significant relation was observed between the presence of sleep disorders and the parent-perceived problems subscale of the BPFAS (*p* = 0.36, Mann–Whitney U test in all cases).

### 3.5. Logistic Regression Analyses

Multivariate analyses using binary logistic regression were performed to identify variables associated with the presence or absence of sleep disorders (dependent variable). The independent variables included in the model were age, sex, ASD severity, presence of associated medical conditions, genetic disorders, and scores on the BPFAS frequency and parent-perceived problems subscales. Multivariate logistic regression indicated that sleep disturbances were significantly and independently associated with ASD severity (*p* = 0.01; OR = 0.23; 95% CI: 0.06–0.77), and BPFAS frequency subscale score (*p* = 0.01; OR = 1.01; 95% CI: 1.01–1.07). The model showed a Cox-Snell R^2^ of 0.18 and a Nagelkerke R^2^ of 0.24, indicating that greater ASD severity and greater feeding difficulties are associated with a higher likelihood of experiencing sleep disorders.

However, there were no significant associations between the presence of sleep disorders with age, sex, presence of medical problems, genetic abnormalities and parent-perceived problems scores (Table 2).

A second logistic regression analysis was performed to identify which items in the BPFAS frequency subscales were associated with the presence of sleep disorders in children and adolescents diagnosed with ASD. Statistically significant associations were found with the following items on the BPFAS: chewing problems (*p* = 0.02; OR = 0.12; 95% CI: 0.02–0.74), comes easily to meals (*p* = 0.01; OR = 0.60; 95% CI: 0.39–0.90), has tantrums during meals (*p* < 0.001; OR = 2.08; 95% CI: 1.21–3.56), has a poor appetite (*p* < 0.001; OR = 2.63; 95% CI: 1.43–4.82), and caregiver perception that the child’s eating pattern affects his or her health (*p* = 0.03; OR = 1.57; 95% CI: 1.03–2.40). The model showed a Cox-Snell R^2^ of 0.23 and a Nagelkerke R^2^ of 0.31. No significant associations were observed with the remaining items (Table 3).

## 4. Discussion

These findings suggest that children and adolescents with greater sleep disturbances tend to present more problematic eating behaviors, especially in aspects related to sleep regulation and the onset of nighttime rest.

We confirmed an increased prevalence of poor sleep quality in ASD youths measured with the Pittsburgh Sleep Quality Index [56], which was also confirmed in previous studies [16,57,58]. Our results demonstrated a statistically significant correlation between sleeping disturbances and autism severity. Participants with the most severe autism had the highest SDSC scores, particularly in the subscales measuring disorders of initiating and maintaining sleep and excessive daytime sleepiness. These results coincide with previous studies linking sleep disorders in ASD to sensory processing abnormalities [59,60], cognitive rigidity [45,61,62], and difficulties with emotional regulation [63,64,65,66,67].

Feeding problems are among the most challenging behavioral issues faced by parents/caregivers of children with ASD [35,68]. However, the underlying mechanism remains unclear. The reasons why some children with ASD have no or minimal feeding problems are also unknown. Several explanations have been proposed, including the possibility that the feeding patterns in children with ASD may not be explained solely by developmental delay but may be due to restricted interests and repetitive behaviors characteristic of the condition [30,38,69,70]. Another possible explanation is that family eating practices may contribute to these problems, either through reduced exposure to a variety of foods [71,72,73] or through inadvertent reinforcement of maladaptive mealtime behaviors [74,75].

Our study identified a significant association between sleep disturbances and feeding disorders in children and adolescents with ASD. This association was strongest for the SDSC subscales measuring disorders of sleep onset and maintenance, sleep arousal disorders and of sleep–wake transition disorder.

We observed that for each increase in BPFAS frequency subscale score, there was a corresponding increase in the risk of having a sleep disorder. To our knowledge, this is the first study to identify specific feeding behaviors as predictors of poor sleep quality in individuals with ASD, namely difficulty chewing, refusal to voluntary attend meals, tantrums during meals, poor appetite, and the caregiver perception that feeding negatively affects the child’s health.

It is plausible that some feeding challenges are related to physiological and behavioral regulation processes, which also affect sleep. Previous studies have explored the relationship between gastrointestinal dysfunction and sleep disorders in ASD, suggesting a possible shared biological mechanism affecting behavior and clinical presentation [41,43,44,48,76,77]. Sensory disturbances can also affect proprioception, so they may have difficulty interpreting hunger signals and therefore maintaining a low appetite [71]. Many children with autism present chronic gastrointestinal disorders, including chronic constipation, diarrhea, nausea, and abdominal pain [78,79]. They also often present selective feeding behaviors, which are associated with low appetite and nutritional problems [38]. It has been established that the presence of gastrointestinal disorders causes low appetite in ASD, generating intestinal dysbiosis that changes the composition of the intestinal microbiota [80,81,82], with a negative impact on tryptophan absorption and negatively affecting melatonin synthesis [83]. The altered microbiota generates a negative impact on sleep quality [84], and vice versa [85,86].

Masticatory dysfunction is one of the most prevalent feeding problems in ASD [87,88,89,90]. Sensory hypersensitivity, particularly oral hypersensitivity, can negatively affect chewing [89,91,92]. These sensory problems may also affect sleep through hypersensitivity to light, sounds or bed linen textures [24,26,93].

In our study, the most common maladaptive behaviors were refusal to attend meals, tantrums during meals, and bedtime resistance, consistent with previous studies [27,44].

Poor appetite has been associated with the development of gastrointestinal disorders, which are also related to excessive daytime sleepiness due to fragmented sleep [42]. We found no significant association between feeding behavioral disorders and ASD severity, in accordance with other studies suggesting that atypical feeding behavior such as food refusal, pickiness, or maladaptive or ritualistic mealtime behavior are not related to cognitive or language ability in children with ASD, but are related to temper tantrums [94,95]. Indeed, children described as having an easy disposition were also more likely to have adequate feeding compared to children who were fussy [34,35,96].

Our findings demonstrate that both greater ASD severity and more pronounced feeding problems are independently associated with an increased risk of sleep disturbances in children and adolescents with ASD. Specific maladaptive mealtime behaviors—such as poor appetite, tantrums during meals, chewing difficulties, refusal to attend meals voluntarily, and caregiver perception that eating negatively impacts the child’s health—may serve as predictors of poor sleep quality. These results underscore the need for integrated screening and early intervention strategies that address both sleep and feeding behaviors in children and adolescents with ASD. Our results may provide some guidance for the design of intervention plans and some progress in understanding ASD and feeding problems. Such interventions may help to improve nutritional status and sleep quality, as well as have beneficial effects on core ASD symptoms and improve overall quality of life for affected children and their families.

Finally, the main limitation of this present study lies in the fact that it was carried out in a single medical center and with a convenience sample, thus making it difficult to extrapolate our results to all ASD populations. The use of convenience sampling limits the generalizability of the findings. Since the participants may not fully represent the broader ASD population, the results should be interpreted with caution. However a priori sample size calculation was reached leading to acceptable findings on this study’s main outcome, at least in the survey’s responders. The absence of this information makes determining the potential for nonresponse bias challenging. The study was based only on self-reported data, which makes it hard to know how right these answers are without observational or objective measures of sleep and feeding habits. Therefore, studies with larger samples with probabilistic sampling are desired in future studies. The study is based on surveys filled out by parents/caregivers who report their subjective experiences with their autistic children. We have not differentiated if they survey respondent is the father or the mother and thus, there may be a gender bias in our results.

There is evidence that there may be differences in perceptions of children’s problems depending on whether fathers or mothers are interviewed, especially in problems related to emotions and anxiety [97], or if there is conflict following a divorce [98].

In a study on children’s sleep in a Spanish population study without ASD [99], there was a high rate of agreement between fathers’ and mothers’ responses but less agreement on the perception of daytime sleepiness and delayed sleep onset. Regarding eating habits, fathers tend to report greater levels of pressure regarding eating than mothers [100].

Two-thirds of our sample were patients with autism degree 2 or 3, with notable communication difficulties, which limits the information available regarding their sleep quality and emotional wellbeing. We have not studied whether medical or psychiatric comorbidities or concomitant medications have an effect on eating or sleep quality.

## 5. Conclusions

The relationship between sleep disorders and feeding disorders in ASD has not been widely studied. This paper provides results that may help understand this relationship and its impact on nuclear autism symptoms and on behavior related to feeding. Our results may provide some guidance for the design of intervention plans and some progress in understanding ASD and feeding problems.

## Figures and Tables

**Table 1:** Clinical and sociodemographic characteristics of the study sample.

Variable	Frequency % and the Mean ± Standard Error of the Mean for Discrete Variables (Range Min–Max)
Age	10.73 ± 0.41 [3,4,5,6,7,8,9,10,11,12,13,14,15,16,17]
Sex	
Male	76% (n = 73)
Female	24% (n = 23)
Level of ASD:	
I	36.5% (n = 35)
II	34.4% (n = 33)
III	29.2% (n = 28)
Medical comorbidities	
Yes	55.2% (n = 53)
No	44.8% (n = 43)
Genetic abnormalities	
Yes	19.8% (n = 19)
No	80.2% (n = 77)
Other psychiatric disorders	
Yes	65.6% (n = 63)
No	34.4% (n = 33)

**Table 2:** Logistic regression: association between sleep disorders and clinical and dietary factors in children and adolescents with ASD.

Independent Variable	Sleep Disorders (Cut-Off Score ≥ 39) OR (95% CI)	*p* Value	Collinearity Statistics	
			Tolerance	Variance Inflation Factor VIF
Age	1.07 (0.95–1.21)	0.21	0.92	1.08
Sex	1.64 (0.58–4.67)	0.34	0.97	1.02
Severity of autism	0.23 (0.06–0.77)	0.01	0.86	1.15
Associated medical conditions	0.99 (0.39–2.51)	0.98	0.88	1.13
Genetic disorders	0.66 (0.20–2.18)	0.50	0.90	1.11
BPFAS—Frequency subscale	1.04 (1.01–1.07)	0.01	0.76	1.31
BPFAS—Perceived Problems	1.02 (0.91–1.14)	0.70	0.74	1.33

**Table 3:** Associations between sleep disorders and feeding disturbances.

Independent Variable	Sleep Disorders (Cut-Off Score ≥ 39) OR (95% CI)	*p* Value	Collinearity Statistics	
			Tolerance	Variance Inflation Factor VIF
Eats fruit	0.72 (0.14–3.67)	0.70	0.89	1.14
Has problem chewing food	0.12 (0.02–0.74)	0.02	0.72	1.38
Enjoys eating	2.97 (0.32–27.01)	0.33	0.59	1.67
Chokes or gags at mealtimes	1.57 (0.94–2.65)	0.08	0.73	1.37
Will try new foods	0.80 (0.55–1.17)	0.26	0.59	1.68
Eats meats	1.02 (0.67–1.55)	0.90	0.72	1.37
Takes longer than 20 min to finish a meal	0.92 (0.66–1.28)	0.64	0.85	1.16
Drinks milk	0.88 (0.67–1.15)	0.35	0.87	1.14
Comes readily to mealtime	0.60 (0.39–0.90)	0.01	0.70	1.41
Eats junk food/snacks but will not eat at mealtime	1.34 (0.93–1.93)	0.11	0.91	1.09
Vomits just before or just after mealtime	1.04 (0.48–2.26)	0.91	0.89	1.12
Eat only ground, strained or soft food	0.92 (0.58–1.44)	0.72	0.72	1.38
Gets up from the table during mealtimes	0.99 (0.72–1.35)	0.96	0.73	1.35
Lets food sit in his/her mouth and does not swallow it	1.49 (0.85–2.60)	0.16	0.62	1.60
Whines or cries at feeding time	1.10 (0.68–1.77)	0.69	0.44	2.22
Eats vegetables	0.94 (0.66–1.33)	0.73	0.71	1.39
Tantrums at mealtimes	2.08 (1.21–3.56)	0.00	0.55	1.80
Eat starches (example, potato, noodles)	0.99 (0.63–1.55)	0.96	0.86	1.15
Has a poor appetite	2.63 (1.43–4.82)	0.00	0.66	1.50
Spits out food	0.94 (0.54–1.65)	0.85	0.67	1.50
Delays eating by talking	0.96 (0.58–1.59)	0.88	0.68	1.46
Would rather drink than eat	0.76 (0.43.1.36)	0.36	0.44	2.27
Refuses to eat but request food immediately after the meal	1.49 (0.82–2.69)	0.18	0.56	1.77
Tries to negotiate what he/she will eat and what he/she will not eat	0.99 (0.68–1.44)	0.96	0.62	1.58
Has required nasal-gastric feeds to maintain proper nutritional status	4.15 (0.59–29.06)	0.15	0.81	1.22
I get frustrated or anxious when feeding my child	0.97 (0.64–1.46)	0.88	0.44	2.23
I coax my child to get him/her to take a bite	1.19 (0.73–1.92)	0.47	0.54	1.84
I use threats to get my child to eat	1.23 (0.63–2.39)	0.53	0.65	1.53
I feel confident my child gets enough to eat	0.67 (0.42–1.07)	0.09	0.65	1.52
I feel confident in my ability to manage my child’s behavior at mealtime	1.63 (0.98–2.71)	0.05	0.70	1.42
If my child does not like what is being served, I do something else	1.04 (0.70–1.54)	0.82	0.69	1.43
When my child refuses to eat, I put the food in his/her mouth by force if necessary	1.97 (0.70–5.52)	0.19	0.83	1.20
I disagree with other adults (example, my spouse, the child’s grandparents) about how to feed my child	0.86 (0.58–1.27)	0.46	0.94	1.06
I feel that my child’s eating pattern hurts his/her general heath	1.57 (1.03–2.40)	0.03	0.67	1.48
I get so angry with my child at mealtimes that it takes me a while to calm down after the meal	1.13 (0.73–1.74)	0.57	0.69	1.44

## Data Availability

The data presented in this study are available upon request with a scientific purpose from the corresponding author.

## References

[1] American Psychiatric Association (2014). DSM-V Manual Estadistico Diagnóstico. Manual Diagnóstico y Estadístico de Los Trastornos Mentales DSM-5.

[2] Zeidan J., Fombonne E., Scorah J., Ibrahim A., Durkin M.S., Saxena S., Yusuf A., Shih A., Elsabbagh M. (2022). Global prevalence of autism: A systematic review update. Autism Res..

[3] Micai M., Fatta L., Gila L., Caruso A., Salvitti T., Fulceri F., Ciaramella A., D’Amico R., Del Giovane C., Bertelli M. (2023). Prevalence of co-occurring conditions in children and adults with autism spectrum disorder: A systematic review and meta-analysis. Neurosci. Biobehav. Rev..

[4] Khachadourian V., Mahjani B., Sandin S., Kolevzon A., Buxbaum J.D., Reichenberg A., Janecka M. (2023). Comorbidities in autism spectrum disorder and their etiologies. Transl. Psychiatry.

[5] Delahaye J., Kovacs E., Sikora D., Hall T.A., Orlich F., Clemons T.E., Van der Weerd E., Glick L., Kuhlthau K. (2014). The relationship between Health-Related Quality of Life and sleep problems in children with Autism Spectrum Disorders. Res. Autism. Spectr. Disord..

[6] Kuhlthau K., Orlich F., Hall T.A., Sikora D., Kovacs E.A., Delahaye J., Clemons T.E. (2010). Health-Related Quality of Life in Children with Autism Spectrum Disorders: Results from the Autism Treatment Network. J. Autism. Dev. Disord..

[7] Reynolds A.M., Malow B.A. (2011). Sleep and Autism Spectrum Disorders. Pediatr. Clin. N. Am..

[8] Richdale A.L., Schreck K.A. (2009). Sleep problems in autism spectrum disorders: Prevalence, nature, & possible biopsychosocial aetiologies. Sleep Med. Rev..

[9] Johnson K.P., Zarrinnegar P. (2021). Autism Spectrum Disorder and Sleep. Child Adolesc. Psychiatr. Clin. N. Am..

[10] Hodge D., Carollo T.M., Lewin M., Hoffman C.D., Sweeney D.P. (2014). Sleep patterns in children with and without autism spectrum disorders: Developmental comparisons. Res. Dev. Disabil..

[11] Schreck K.A., Richdale A.L. (2020). Sleep problems, behavior, and psychopathology in autism: Inter-relationships across the lifespan. Curr. Opin. Psychol..

[12] Schwichtenberg A.J., Janis A., Lindsay A., Desai H., Sahu A., Kellerman A., Chong P., Abel E., Yatcilla J. (2022). Sleep in Children with Autism Spectrum Disorder: A Narrative Review and Systematic Update. Curr. Sleep Med. Rep..

[13] Cohen S., Conduit R., Lockley S.W., Rajaratnam S.M., Cornish K.M. (2014). The relationship between sleep and behavior in autism spectrum disorder (ASD): A review. J. Neurodev. Disord..

[14] Schreck K.A., Mulick J.A., Smith A.F. (2004). Sleep problems as possible predictors of intensified symptoms of autism*1. Res. Dev. Disabil..

[15] Micsinszki S.K., Ballantyne M., Cleverley K., Green P., Brennenstuhl S., Stremler R. (2023). Examining factors associated with sleep quality in parents of children 4–10 years with autism spectrum disorder. Disabil. Rehabil..

[16] Tudor M.E., Hoffman C.D., Sweeney D.P. (2012). Children with Autism. Focus Autism Other Dev. Disabil..

[17] Bianca B., Silvia G., Elisa F., Deny M., Giovanni V., Lino N., Stefano V. (2024). Insomnia in Children with Autism Spectrum Disorder: A Cross-Sectional Study on Clinical Correlates and Parental Stress. J. Autism Dev. Disord..

[18] Ji Q., Li S.J., Zhao J.B., Xiong Y., Du X.H., Wang C.X., Lu L., Tan J., Zhu Z. (2023). Genetic and neural mechanisms of sleep disorders in children with autism spectrum disorder: A review. Front Psychiatry.

[19] Ritvo E.R., Ritvo R., Yuwiler A., Brothers A., Freeman B.J., Plotkin S. (1993). Elevated daytime melatonin concentrations in autism: A pilot study. Eur. Child Adolesc. Psychiatry.

[20] Tordjman S., Anderson G.M., Bellissant E., Botbol M., Charbuy H., Camus F., Graignic R., Kermarrec S., Fougerou C., Cohen D. (2012). Day and nighttime excretion of 6-sulphatoxymelatonin in adolescents and young adults with autistic disorder. Psychoneuroendocrinology.

[21] Melke J., Goubran Botros H., Chaste P., Betancur C., Nygren G., Anckarsäter H., Rastam M., Ståhlberg O., Gillberg I., Delorme R. (2008). Abnormal melatonin synthesis in autism spectrum disorders. Mol. Psychiatry.

[22] Pinato L., Galina Spilla C.S., Markus R.P., da Silveira Cruz-Machado S. (2020). Dysregulation of Circadian Rhythms in Autism Spectrum Disorders. Curr. Pharm. Des..

[23] Tzischinsky O., Meiri G., Manelis L., Bar-Sinai A., Flusser H., Michaelovski A., Zivan O., Ilan M., Faroy M., Menashe I. (2018). Sleep disturbances are associated with specific sensory sensitivities in children with autism. Mol. Autism..

[24] Lane S.J., Leão M.A., Spielmann V. (2022). Sleep, Sensory Integration/Processing, and Autism: A Scoping Review. Front. Psychol..

[25] Mazurek M.O., Petroski G.F. (2015). Sleep problems in children with autism spectrum disorder: Examining the contributions of sensory over-responsivity and anxiety. Sleep Med..

[26] Souders M.C., Zavodny S., Eriksen W., Sinko R., Connell J., Kerns C., Schaaf R., Pinto-Martin R. (2017). Sleep in Children with Autism Spectrum Disorder. Curr. Psychiatry Rep..

[27] Hunter J.E., McLay L.K., France K.G., Blampied N.M. (2021). Sleep and stereotypy in children with autism: Effectiveness of function-based behavioral treatment. Sleep Med..

[28] Bandini L.G., Anderson S.E., Curtin C., Cermak S., Evans E.W., Scampini R., Maslin M., Must A. (2010). Food Selectivity in Children with Autism Spectrum Disorders and Typically Developing Children. J. Pediatr..

[29] Cermak S.A., Curtin C., Bandini L.G. (2010). Food Selectivity and Sensory Sensitivity in Children with Autism Spectrum Disorders. J. Am. Diet. Assoc..

[30] Ferrara R., Iovino L., Ricci L., Avallone A., Latina R., Ricci P. (2025). Food selectivity and autism: A systematic review. World J. Clin. Pediatr..

[31] Marí-Bauset S., Zazpe I., Mari-Sanchis A., Llopis-González A., Morales-Suárez-Varela M. (2014). Food Selectivity in Autism Spectrum Disorders. J. Child. Neurol..

[32] Molina-López J., Leiva-García B., Planells E., Planells P. (2021). Food selectivity, nutritional inadequacies, and mealtime behavioral problems in children with autism spectrum disorder compared to neurotypical children. Int. J. Eat. Disord..

[33] Page S.D., Souders M.C., Kral T.V.E., Chao A.M., Pinto-Martin J. (2022). Correlates of Feeding Difficulties Among Children with Autism Spectrum Disorder: A Systematic Review. J. Autism Dev. Disord..

[34] Baraskewich J., von Ranson K.M., McCrimmon A., McMorris C.A. (2021). Feeding and eating problems in children and adolescents with autism: A scoping review. Autism.

[35] van Dijk M.W.G., Buruma M.E., Blijd-Hoogewys E.M.A. (2021). Detecting Feeding Problems in Young Children with Autism Spectrum Disorder. J. Autism Dev. Disord..

[36] Sharp W.G., Berry R.C., McCracken C., Nuhu N.N., Marvel E., Saulnier C.A., Klin A., Jones W., Jaquess D. (2013). Feeding Problems and Nutrient Intake in Children with Autism Spectrum Disorders: A Meta-analysis and Comprehensive Review of the Literature. J. Autism Dev. Disord..

[37] Sammels O., Karjalainen L., Dahlgren J., Wentz E. (2022). Autism Spectrum Disorder and Obesity in Children: A Systematic Review and Meta-Analysis. Obes. Facts..

[38] Esposito M., Mirizzi P., Fadda R., Pirollo C., Ricciardi O., Mazza M., Valenti M. (2023). Food Selectivity in Children with Autism: Guidelines for Assessment and Clinical Interventions. Int. J. Environ. Res. Public Health.

[39] Guller B., Yaylaci F. (2024). Eating and sleep problems, related factors, and effects on the mental health of the parents in children with autism spectrum disorder. Int. J. Dev. Disabil..

[40] Allen S.L., Smith I.M., Duku E., Vaillancourt T., Szatmari P., Bryson S., Fombonne E., Volden J., Waddell C., Zwaigenbaum L. (2015). Behavioral Pediatrics Feeding Assessment Scale in Young Children with Autism Spectrum Disorder: Psychometrics and Associations with Child and Parent Variables. J. Pediatr. Psychol..

[41] Orr W.C., Fass R., Sundaram S.S., Scheimann A.O. (2020). The effect of sleep on gastrointestinal functioning in common digestive diseases. Lancet Gastroenterol. Hepatol..

[42] Jansen E.C., Dunietz G.L., Felt B.T., O’Brien L.M. (2018). Sleep and Gastrointestinal Symptoms in a Community-Based Survey of Children. Clin. Pediatr..

[43] Yang X.L., Liang S., Zou M.Y., Sun C.H., Han P.P., Jiang X.T., Xia W., Wu L. (2018). Are gastrointestinal and sleep problems associated with behavioral symptoms of autism spectrum disorder?. Psychiatry Res..

[44] Bresciani G., Da Lozzo P., Lega S., Bramuzzo M., Di Leo G., Dissegna A., Colonna V., Barbi E., Carrozzi M., Devescovi R. (2023). Gastrointestinal Disorders and Food Selectivity: Relationship with Sleep and Challenging Behavior in Children with Autism Spectrum Disorder. Children.

[45] Passarini S., Parisi M., Guerrera S., Lazzaro G., Costanzo F., Menghini D., Vicari S., Fucà E. (2025). Exploring the association between sleep disturbances and repetitive behaviors in autistic children and adolescents: A systematic review. Sleep Med..

[46] Muller C.L., Anacker A.M.J., Veenstra-VanderWeele J. (2016). The serotonin system in autism spectrum disorder: From biomarker to animal models. Neuroscience.

[47] Israelyan N., Margolis K.G. (2018). Serotonin as a link between the gut-brain-microbiome axis in autism spectrum disorders. Pharmacol. Res..

[48] Ding W., Xu Y., Ding W., Tang Q., Zhang B., Yuan Y., Jin J. (2025). Research progress on melatonin, 5-HT, and orexin in sleep disorders of children with autism spectrum disorder. Biomol. Biomed..

[49] Marcos P., Coveñas R. (2021). Involvement of the Orexinergic System in Feeding. Appl. Sci..

[50] Kraemer H.C. (2006). Correlation coefficients in medical research: From product moment correlation to the odds ratio. Stat. Methods Med. Res..

[51] Mukaka M.M. (2012). Statistics corner: A guide to appropriate use of correlation coefficient in medical research. Malawi Med. J..

[52] Crist W., Mcdonnell P., Beck M., Gillespie C.T., Barrett P., Mathews J. (1994). Behavior at Mealtimes and the Young Child with Cystic Fibrosis. J. Dev. Behav. Pediatr..

[53] López López M.C. (2022). Dificultades de Alimentación en Edad Pediátrica: Traducción, Ampliación y Validación de la “Behavioral Pediatrics Feeding Assessment Scale”. Universidad de Valladolid. https://uvadoc.uva.es/handle/10324/54297.

[54] Bruni O., Ottaviano S., Guidetti V., Romoli M., Innocenzi M., Cortesi F., Giannotti F. (1996). The Sleep Disturbance Scale for Children (SDSC) Construct ion and validation of an instrument to evaluate sleep disturbances in childhood and adolescence. J. Sleep Res..

[55] Pagerols M., Bosch R., Prat R., Pagespetit È., Cilveti R., Chaparro N., Esteve A., Casas M. (2023). The Sleep Disturbance Scale for Children: Psychometric properties and prevalence of sleep disorders in Spanish children aged 6–16 years. J Sleep Res..

[56] Fuentes-Albero M., Mafla-España M.A., Martínez-Raga J., Cauli O. (2024). Salivary IL-1 Beta Level Associated with Poor Sleep Quality in Children/Adolescents with Autism Spectrum Disorder. Pediatr. Rep..

[57] Miner S., McVoy M., Damato E. (2023). Evaluation of the Relationship of Sleep Disturbances to Severity and Common Behaviors in Autism Spectrum Disorder. Res. Sq..

[58] Mazzone L., Postorino V., Siracusano M., Riccioni A., Curatolo P. (2018). The Relationship between Sleep Problems, Neurobiological Alterations, Core Symptoms of Autism Spectrum Disorder, and Psychiatric Comorbidities. J. Clin. Med..

[59] Raj S.D.V., Umaiorubagam G.S. (2025). Relationship Between Sensory Processing and Sleep in Children with Autism Spectrum Disorder. Chronobiol. Med..

[60] Iwasaki S., Yoshimura Y., Hasegawa C., Tanaka S., Ikeda T., Yaoi K., Hirosawa T., Kikuchi M. (2025). Sleep problems and sensory features in children with low-average cognitive abilities and autism spectrum disorder. Sci. Rep..

[61] Berenguer C., Lacruz-Pérez I., Rosa E., de Stasio S., Choque-Olsson N. (2024). The implication of sleep disturbances on daily executive functioning and learning problems in children with autism without intellectual disability. Res. Autism Spectr. Disord..

[62] Lenker K.P., Li Y., Fernandez-Mendoza J., Mayes S.D., Calhoun S.L. (2025). Autism Spectrum Disorder Phenotypes Based on Sleep Dimensions and Core Autism Symptoms. J. Autism Dev. Disord..

[63] Sommers L., Papadopoulos N., Fuller-Tyszkiewicz M., Sciberras E., McGillivray J., Howlin P., Rinehart N. (2025). The Connection Between Sleep Problems and Emotional and Behavioural Difficulties in Autistic Children: A Network Analysis. J. Autism Dev. Disord..

[64] Richdale A.L., Shui A.M., Lampinen L.A., Katz T. (2024). Sleep disturbance and other co-occurring conditions in autistic children: A network approach to understanding their inter-relationships. Autism Res..

[65] Schreck K.A., Mulick J.A. (2000). Parental Report of Sleep Problems in Children with Autism. J. Autism Dev. Disord..

[66] Shui A.M., Katz T., Malow B.A., Mazurek M.O. (2018). Predicting sleep problems in children with autism spectrum disorders. Res. Dev. Disabil..

[67] Coury D. (2010). Medical treatment of autism spectrum disorders. Curr. Opin. Neurol..

[68] Kral T.V.E., Souders M.C., Tompkins V.H., Remiker A.M., Eriksen W.T., Pinto-Martin J.A. (2015). Child Eating Behaviors and Caregiver Feeding Practices in Children with Autism Spectrum Disorders. Public Health Nurs..

[69] Riccio M.P., Marino M., Garotti R., Tassiello A., Maffettone V., Pezone M., Bravaccio C. (2025). Food selectivity in Autism Spectrum Disorder: Implications of eating, sensory and behavioural profile. Front. Psychiatry.

[70] Doğar Karaca B., Durcan G., Sandikci T., Cokugras H., Doğangün B. (2025). The relationship between sensory sensitivity and eating problems in children with autism spectrum disorder. Res. Autism.

[71] Schreck K.A., Williams K. (2006). Food preferences and factors influencing food selectivity for children with autism spectrum disorders. Res. Dev. Disabil..

[72] Gent V., Marshall J., Weir K., Trembath D. (2025). Caregiver perspectives regarding the impact of feeding difficulties on mealtime participation for primary school-aged autistic children and their families. Int. J. Speech Lang. Pathol..

[73] Ausderau K.K., John B.M.S., Al-Heizan M.O., Dammann C., Chaudoir S., Sideris J. (2024). Factor analysis of the feeding and eating in AutiSm Together Assessment. Res. Autism Spectr. Disord..

[74] Castro K., Frye R.E., Silva E., Vasconcelos C., Hoffmann L., Riesgo R., Vaz J. (2024). Feeding-Related Early Signs of Autism Spectrum Disorder: A Narrative Review. J. Pers. Med..

[75] Levin L., Carr E.G. (2001). Food Selectivity and Problem Behavior in Children with Developmental Disabilities. Behav. Modif..

[76] Holingue C., Pfeiffer D., Ludwig N.N., Reetzke R., Hong J.S., Kalb L.G., Landa R. (2023). Prevalence of gastrointestinal symptoms among autistic individuals, with and without co-occurring intellectual disability. Autism Res..

[77] Bonmatí-Carrión M.Á., Rol M.A. (2023). Melatonin as a Mediator of the Gut Microbiota–Host Interaction: Implications for Health and Disease. Antioxidants.

[78] Wang J., Ma B., Wang J., Zhang Z., Chen O. (2022). Global prevalence of autism spectrum disorder and its gastrointestinal symptoms: A systematic review and meta-analysis. Front. Psychiatry.

[79] Al-Beltagi M., Saeed N.K., Bediwy A.S., Elbeltagi R., Alhawamdeh R. (2023). Role of gastrointestinal health in managing children with autism spectrum disorder. World J. Clin. Pediatr..

[80] Gonçalves C.L., Doifode T., Rezende V.L., Costa M.A., Rhoads J.M., Soutullo C.A. (2024). The many faces of microbiota-gut-brain axis in autism spectrum disorder. Life Sci..

[81] Han H., Yi B., Zhong R., Wang M., Zhang S., Ma J., Yin Y., Yin J., Chen L., Zhang H. (2021). From gut microbiota to host appetite: Gut microbiota-derived metabolites as key regulators. Microbiome.

[82] Moran G.W., Thapaliya G. (2021). The Gut–Brain Axis and Its Role in Controlling Eating Behavior in Intestinal Inflammation. Nutrients.

[83] Aziz-Zadeh L., Ringold S.M., Jayashankar A., Kilroy E., Butera C., Jacobs J.P., Tanartkit S., Mahurkar-Joshi S., Bhatt R., Dapretto M. (2025). Relationships between brain activity, tryptophan-related gut metabolites, and autism symptomatology. Nat. Commun..

[84] Hua X., Zhu J., Yang T., Guo M., Li Q., Chen J., Li T. (2020). The Gut Microbiota and Associated Metabolites Are Altered in Sleep Disorder of Children with Autism Spectrum Disorders. Front. Psychiatry.

[85] Wang L., Wang B., Wu C., Wang J., Sun M. (2023). Autism Spectrum Disorder: Neurodevelopmental Risk Factors, Biological Mechanism, and Precision Therapy. Int. J. Mol. Sci..

[86] Sen P., Molinero-Perez A., O’Riordan K.J., McCafferty C.P., O’Halloran K.D., Cryan J.F. (2021). Microbiota and sleep: Awakening the gut feeling. Trends Mol. Med..

[87] Nadon G., Feldman D.E., Dunn W., Gisel E. (2011). Mealtime problems in children with Autism Spectrum Disorder and their typically developing siblings: A comparison study. Autism.

[88] Şahan A.K., Öztürk N., Demir N., Karaduman A.A., Arslan S.S. (2021). A Comparative Analysis of Chewing Function and Feeding Behaviors in Children with Autism. Dysphagia.

[89] Shalini M., Swapna N. (2025). The Chewing Challenge: Linking Oral Motor and Oral Sensory Skills to Feeding Difficulties in Autism. J. Indian Speech Lang. Hear. Assoc..

[90] Leader G., Tuohy E., Chen J.L., Mannion A., Gilroy S.P. (2020). Feeding Problems, Gastrointestinal Symptoms, Challenging Behavior and Sensory Issues in Children and Adolescents with Autism Spectrum Disorder. J. Autism Dev. Disord..

[91] Thompson K., Wallisch A., Nowell S., Meredith J., Boyd B. (2023). Short report: The role of oral hypersensitivity in feeding behaviors of young autistic children. Autism.

[92] Raj N.M., Veena K.D., Rajashekhar B., Sreelakshmi A.C.A. (2024). Oral Sensory Issues with Feeding and Communication Skills in Autistic Children. Adv. Neurodev. Disord..

[93] Mazurek M.O., Sohl K. (2016). Sleep and Behavioral Problems in Children with Autism Spectrum Disorder. J. Autism Dev. Disord..

[94] Aponte C.A., Romanczyk R.G. (2016). Assessment of feeding problems in children with autism spectrum disorder. Res. Autism Spectr. Disord..

[95] Dominick K.C., Davis N.O., Lainhart J., Tager-Flusberg H., Folstein S. (2007). Atypical behaviors in children with autism and children with a history of language impairment. Res. Dev. Disabil..

[96] Williams P.G., Dalrymple N., Neal J. (2000). Eating habits of children with autism. Pediatr. Nurs..

[97] Tackett J.L. (2011). Parent Informants for Child Personality: Agreement, Discrepancies, and Clinical Utility. J. Pers. Assess..

[98] Brocker S.A., Steinbach A., Augustijn L. (2024). Parent-child Discrepancies in Reporting Children’s Mental Health: Do Physical Custody Arrangements in Post-separation Families Matter?. Child Indic. Res..

[99] Becker S.P., Isaacson P.A., Servera M., Sáez B., Burns G.L. (2017). Mother–father agreement and one-year stability of children’s sleep functioning. Sleep Med..

[100] Patel P., Samant A., Del Rosario K., Vitolins M.Z., Skelton J.A., Ip E.H., Lucas C., Brown C. (2024). Differences in maternal and paternal pressure to eat and perception of household responsibilities. PLoS ONE.

